# Mechanical Performance of Uncompatibilized Recycled Polypropylene Biocomposites Filled with Corn, Banana, and Barley Agro-Industrial Residue Fibers

**DOI:** 10.3390/polym18111384

**Published:** 2026-06-02

**Authors:** Juan Fernando García, Juan Diego Febres

**Affiliations:** Departamento de Química y Producción, Universidad Técnica Particular de Loja, Loja 110107, Ecuador; jdfebres@utpl.edu.ec

**Keywords:** recycled polypropylene, natural fiber composites, uncompatibilized composites, mechanical characterization, loading-mode-dependent behavior, circular economy, agro-industrial residue valorization, corn stover, banana pseudostem, barley husk

## Abstract

Recycled polypropylene (rPP) biocomposites represent a convergent strategy for plastic waste valorization and agro-industrial residue reutilization. This study quantifies tensile, flexural, and compressive performance (ASTM D638, D790, D695) of rPP biocomposites incorporating raw corn stover (*Zea mays*), banana pseudostem (*Musa* spp.), and barley residue (*Hordeum vulgare*) fibers at 10, 20, and 30 wt%, processed by single-screw extrusion and compression molding without compatibilizer. Two-way ANOVA with Tukey HSD post hoc analysis (α = 0.05) evaluated effects of fiber type and concentration. Tensile strength declined monotonically across all systems, from 24.9 MPa (neat rPP) to 7.9 MPa at 30 wt% banana fiber. Corn fiber exhibited exceptional tensile concentration stability (only −11% across the full range) and the best flexural retention at 10 wt% (36.6 MPa, 79% of neat rPP). A performance plateau was identified at 20 wt% under both tensile and flexural loading, beyond which further addition produced no significant reduction. Under compression, fiber type exerted its largest statistical effect (F = 81.231), all three systems were mutually distinguishable, and no plateau was observed. These results establish a loading-mode-resolved mechanical baseline for uncompatibilized rPP biocomposites, with corn fiber at 10–20 wt% as the most versatile formulation across all loading modes.

## 1. Introduction

Polypropylene (PP) accounts for approximately 16% of global plastic consumption [1], and end-of-life PP waste has become a priority target in circular economy policy frameworks. Mechanical recycling—converting post-consumer PP into recycled rPP resin—remains the most industrially viable route owing to its technological maturity and low capital requirements, although it entails thermo-mechanical chain degradation and potential incompatibility in heterogeneous waste streams [2]. Chemical recycling routes, including pyrolysis and gasification, offer complementary flexibility for mixed-resin fractions but continue to face cost and scalability constraints [3,4]. The growing interest in combining recycled thermoplastics with lignocellulosic reinforcements as a convergent strategy for plastic waste valorization and biomass reutilization has been documented in recent reviews [5,6].

Incorporating lignocellulosic agro-industrial residues as fillers in rPP matrices addresses two distinct waste streams simultaneously while conferring potential mechanical and thermal benefits. Natural fibers offer competitive specific properties, low density, wide global availability, and biodegradability relative to conventional synthetic fillers [7,8], and recent work has demonstrated the technical feasibility of reinforcing recycled polypropylene with natural fibers such as hemp through melt-processing routes [9]. However, fiber–matrix adhesion is the decisive variable controlling composite performance: the hydrophobic rPP surface is inherently incompatible with hydrophilic cellulosic fibers, promoting interfacial debonding under mechanical loading [10,11]. Maleic anhydride-grafted polypropylene (MAPP) is the most widely employed coupling agent to bridge this incompatibility [12,13], but reactive compatibilization increases formulation cost and process complexity, limiting adoption in cost-sensitive, high-volume applications.

Corn stover, banana pseudostem, and barley husk residues represent abundant agro-industrial by-products in Andean agricultural regions. Corn fibers have demonstrated favorable concentration stability in PP composites due to their aspect ratio and resistance to agglomeration under low-shear processing conditions [11]; banana pseudostem provides moderate reinforcement capacity, though its high hemicellulose content and hydrophilicity impose particular interfacial constraints in hydrophobic matrices that intensify with increasing fiber fraction [14,15,16]; barley husk fibers present a distinctive surface chemistry—a natural coating of fat and protein molecules—that limits inter-fiber agglomeration in thermoplastic matrices, with documented mechanical performance comparable to or exceeding that of softwood-derived fillers in PP systems [13]. Despite this individual body of evidence, a systematic comparative assessment of these three fiber types within a single uncompatibilized rPP matrix, evaluated across tensile, flexural, and compressive loading simultaneously, has not been reported in the literature.

Critically, the natural fiber–PP composite literature has historically focused on tensile, flexural, and impact characterization. Landmark reviews of biocomposites filled with natural fibers—including the comprehensive surveys by Faruk et al. [17] and Pickering et al. [7]—evaluate mechanical performance primarily in terms of these three loading modes, and compressive behavior is only occasionally reported in primary experimental studies of uncompatibilized NF–PP systems [18]. This pattern is consistent with a widespread implicit assumption that fiber type effects are attenuated under compression relative to tensile loading, where failure is more directly governed by fiber–matrix interfacial adhesion. To the authors’ knowledge, this assumption has not been rigorously tested across multiple fiber types and loading levels simultaneously. Moreover, the existence of statistically validated concentration thresholds—beyond which additional fiber loading produces no measurable strength reduction—has not been documented for uncompatibilized natural fiber–recycled PP systems under multiple loading modes, despite its direct relevance to material specification for non-structural applications where multiple loading conditions coexist.

Environmental considerations provide additional motivation. Open-field burning of crop residues remains a widespread practice in Andean agricultural regions, including Ecuador, where it contributes to particulate matter emissions and soil nutrient loss [19]. Global reviews of agricultural burning confirm that these emissions include ozone precursors with documented adverse effects on air quality and human health [20]. Diverting corn stover, banana pseudostem, and barley residues from open burning into composite formulations therefore addresses a dual environmental objective: reducing atmospheric emissions at the source while valorizing an underutilized biomass stream within a circular economy framework. This approach is consistent with Ecuador’s national strategies for solid waste reduction and sustainable agricultural development [21].

This study addresses the following specific gap: what are the mechanical tolerance limits of uncompatibilized rPP biocomposites filled with three distinct local agro-industrial fibers across tensile, flexural, and compressive loading regimes, and does the relative influence of fiber type versus fiber concentration vary systematically with loading mode? The objectives are: (i) to characterize and statistically compare tensile, flexural, and compressive properties of rPP biocomposites filled with raw corn stover, banana pseudostem, and barley residue fibers at 10, 20, and 30 wt%; (ii) to determine the independent and interactive effects of fiber type and concentration via two-way ANOVA and Tukey HSD post hoc analysis; and (iii) to establish loading-mode-resolved composition guidelines for cost-effective, circular non-structural applications.

## 2. Materials and Methods

### 2.1. Raw Materials

Post-consumer rPP was sourced from segregated plastic waste (food trays and containers) in the province of Loja, southern Ecuador, between April 2022 and May 2022. Material identification was performed by visual inspection of the resin identification code, retaining only items marked as resin code 5 (PP) according to ASTM D7611/D7611M-13 [22]. The use of post-consumer rather than virgin polypropylene was deliberate, as the present study aims to evaluate the mechanical performance achievable from locally available waste streams without industrial supply chains.

The three lignocellulosic fibers used in this study—corn stover (*Zea mays*), banana pseudostem (*Musa* spp.), and barley husk (*Hordeum vulgare*)—were collected manually from rural farms in the province of Loja, southern Ecuador, during May 2022. The selection of these three residues reflects their abundance in Andean agricultural regions, where they are commonly discarded or openly burned [19,20].

No coupling agents, surface treatments, or chemical compatibilizers were employed, to isolate the effect of intrinsic fiber–matrix adhesion on composite performance.

### 2.2. Material Preparation

rPP was manually cleaned, de-labeled, washed with tap water, and air-dried on absorbent paper for 48 h before shear-milling to particle sizes of 3–5 mm. Natural fibers were roller-pressed and subsequently milled to homogenize fiber morphology, washed to remove surface impurities, oven-dried at 60 °C to a moisture content below 5%, and classified using a vibrating sieve shaker operated for 5 min per cycle, equipped with M10 (2 mm), M18 (1 mm), M35 (0.5 mm), and M45 (0.355 mm) mesh screens. The fiber fraction retained between the M10 and M18 sieves was selected for composite formulation, corresponding to a nominal particle size range of 1–2 mm. The same classification procedure and size fraction were used for all three fiber types. No chemical or mechanical surface modification was applied to the fibers, to preserve the uncompatibilized test condition.

### 2.3. Composite Formulation

Formulations were prepared at fiber mass fractions of 0 wt% (control), 10 wt%, 20 wt%, and 30 wt%. Total batch mass was calculated from mold volume to ensure accurate mass proportions between the polymer matrix and the fiber phase across all conditions.

Fiber loadings of 10, 20, and 30 wt% were selected to cover the experimentally relevant compositional range for natural fiber–polypropylene composites, consistent with values reported in the literature [7,11,13]. Loadings below 10 wt% were not evaluated, as the literature suggests their mechanical effect tends to be marginal in compression-molded systems [7]; loadings above 30 wt% were excluded due to the substantial processing difficulties associated with increased melt viscosity and poor fiber wetting, which become particularly limiting when no compatibilizer is used [7].

### 2.4. Extrusion Processing

Blends were processed in a single-screw laboratory extruder (screw diameter: 25 mm; L/D = 16) equipped with an INVT GOODDRIVE 10 frequency inverter at 20 rpm. The temperature profile was 165 °C (feed zone), 170 °C (transition zone), and 180 °C (die), yielding a mean barrel temperature of 172 °C. This profile was deliberately kept below the conventional processing window for virgin polypropylene, which is typically reported in the range of 220–280 °C [23], in order to minimize thermal degradation of the lignocellulosic phase. Lignocellulosic fibers begin to undergo measurable thermal degradation above approximately 200 °C. Among the three main biopolymer components of lignocellulosic fibers, hemicellulose exhibits the lowest onset temperature of thermal decomposition (200–300 °C) and is the first to degrade; cellulose decomposes in a narrower window around 300–400 °C; and lignin undergoes the broadest and most thermally stable degradation process, beginning at temperatures as low as 200 °C and with significant decomposition continuing up to approximately 400–500 °C due to its complex polyaromatic structure [24]. In experimental studies of PP–cellulose fiber composites, melt temperatures above approximately 256 °C have been shown to cause measurable fiber length reduction and loss of mechanical properties [25]. Maintaining the barrel temperature at 172 °C—well below both the standard PP processing window and the onset of significant fiber degradation—reduces the risk of fiber scission, volatile release, and discoloration during melt compounding. After extrusion, the material was cooled to ambient temperature, pelletized, and stored under dry conditions prior to molding.

### 2.5. Compression Molding

Specimens were fabricated using a hydraulic hot press at 172 °C with a holding time of 12 min. The mold consisted of an open female cavity without a matching male plug; consequently, the press platens contacted the upper surface of the material without volumetric confinement. The estimated contact pressure—calculated from the piston force divided by the mold cavity area—was approximately 45 kPa, which is substantially lower than the 2–20 MPa range commonly reported for compression molding of natural fiber–polymer composites in the literature, particularly under matched-die conditions [26,27].

This low effective pressure is a direct consequence of the open-cavity mold configuration rather than a deliberately selected processing parameter. As a result, the consolidation conditions in this study are more representative of contact hot-press molding than of conventional matched-die compression molding, which may result in higher residual porosity and weaker fiber–matrix consolidation than would be achieved under confined, high-pressure processing. This processing context should be considered when comparing the absolute mechanical values reported here with those from studies employing conventional compression or injection molding. Mold geometry conformed to ASTM D638 (Type IV dog-bone for tensile) [28], ASTM D790 (rectangular bar for flexural) [29], and ASTM D695 (cylinder for compressive) [30]. Nominal specimen dimensions are summarized in Table 1.

### 2.6. Mechanical Testing

All tests were performed on a Shimadzu AGS-X universal testing machine (Shimadzu Corporation, Kyoto, Japan; 5 kN load cell; Trapezium X^®^ software 2021 v1.5.6) with five replicate specimens per condition (*n* = 5). Tensile tests followed ASTM D638-03 at 5.5 mm/min. Flexural tests used three-point bending (span/depth = 16:1) at a crosshead speed of 1.52 mm/min, calculated per ASTM D790-03 using an outer fiber strain rate Z = 0.1 mm/mm/min (R = ZL^2^/6d). Compressive tests followed ASTM D695-02a at 1.3 mm/min with specimens centered between parallel metal platens. Force and displacement channels were zeroed before each test.

**Figure 1:**
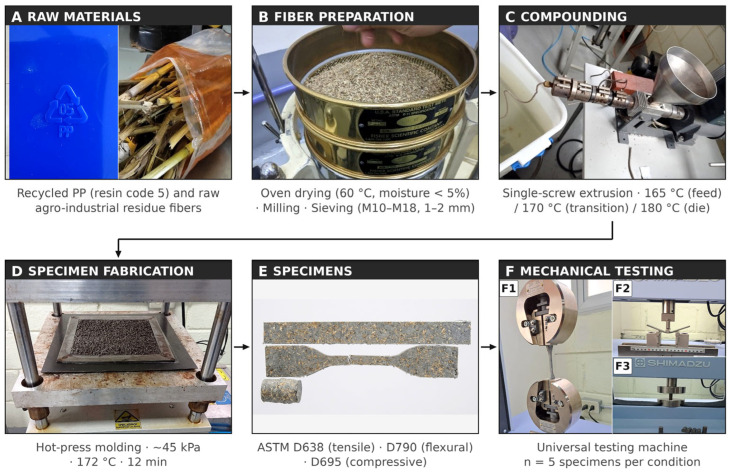
Experimental workflow for the fabrication and mechanical testing of rPP–natural fiber biocomposites. (**A**) Raw materials: recycled polypropylene (resin code 5) and agro-industrial residue fibers. (**B**) Fiber preparation: oven drying (60 °C, moisture content below 5%), milling, and classification by vibrating sieve (M10–M18 fraction, 1–2 mm nominal size). (**C**) Melt compounding via single-screw extrusion (165/170/180 °C feed/transition/die). (**D**) Specimen fabrication by contact hot-press molding (~45 kPa, 172 °C, 12 min). (**E**) Test specimens conforming to ASTM D638 (tensile), D790 (flexural), and D695 (compressive). (**F**) Mechanical testing on a Shimadzu AGS-X universal testing machine: (F1) tensile, (F2) flexural three-point bending, and (F3) compressive loading configurations (*n* = 5 per condition).

Figure 1 provides a visual overview of the complete experimental workflow, from raw material sourcing through mechanical testing.

### 2.7. Statistical Analysis

Descriptive statistics (mean, standard deviation) were computed in Microsoft Excel^®^ 2023 v16.80 (Microsoft Corporation, Redmond, WA, USA). A two-factor full-factorial design was adopted to evaluate the independent and combined effects of fiber type (three levels: banana, corn, barley) and fiber concentration (four levels: 0, 10, 20, 30 wt%) on the mechanical response variables, following standard procedures for factorial experiments in materials research [31]. Five replicate specimens were tested per experimental condition (*n* = 5), consistent with the minimum replication recommended by the applicable ASTM standards [28,29,30]. Two-way ANOVA (factors: fiber type, fiber concentration, and their interaction; α = 0.05) was applied to each mechanical response variable in IBM SPSS^®^ Statistics v26 (IBM Corp., Armonk, NY, USA). Prior to ANOVA, normality of residuals was assessed using the Shapiro–Wilk test and homogeneity of variances was evaluated using Levene’s test; both assumptions were satisfied at α = 0.05. Tukey’s HSD post hoc test was applied to identify statistically significant pairwise differences wherever ANOVA returned a significant main effect or interaction [31].

## 3. Results

### 3.1. Tensile Properties

Table 2 presents the mean tensile strength and maximum elongation at break for neat recycled polypropylene (rPP, control) and for all biocomposite formulations incorporating banana pseudostem, corn stover, and barley residue fibers at 10, 20, and 30 wt%. Results correspond to the mean of five specimens per experimental condition, selected on the basis of data consistency; specimens exhibiting atypical failure modes or load–displacement discontinuities were excluded prior to analysis.

Neat rPP exhibited a tensile strength of 24.91 ± 0.72 MPa with an elongation at a break of 2.75%. The incorporation of natural fibers produced a systematic reduction in both tensile strength and maximum elongation across all formulations, the magnitude of which depended on fiber type and loading level.

Banana fiber composites exhibited the steepest tensile strength decline, falling from 17.32 ± 0.21 MPa at 10 wt% (−30% relative to control) to 7.86 ± 0.75 MPa at 30 wt% (−68%). Corn fiber composites showed markedly different behavior: tensile strength declined only from 14.56 ± 0.42 MPa at 10 wt% to 12.96 ± 1.30 MPa at 30 wt%, a total reduction of 11% across the full loading range. Barley fiber composites showed an intermediate response, declining from 14.61 ± 1.95 MPa at 10 wt% to 9.33 ± 0.73 MPa at 30 wt% (−36%).

**Figure 2:**
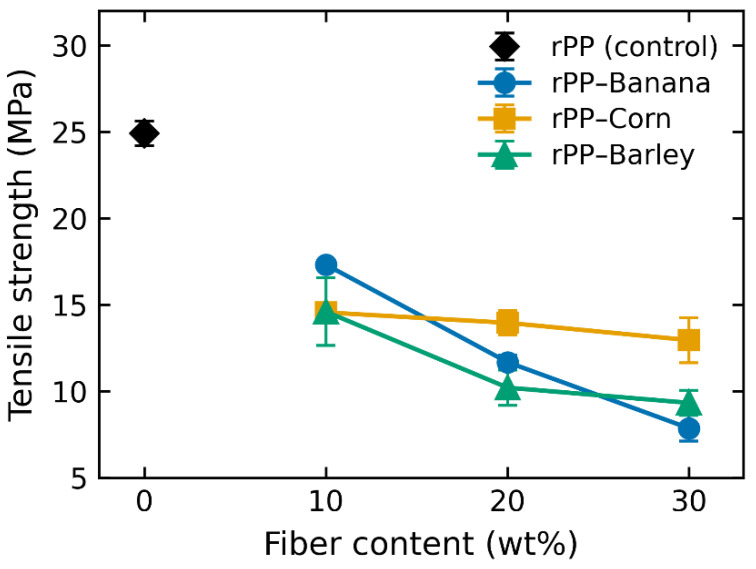
Tensile strength of recycled polypropylene (rPP) and rPP–natural fiber biocomposites as a function of fiber content. Data points represent the mean of five specimens; error bars indicate one standard deviation. The rPP control (filled diamond, 0 wt%) is shown for reference.

Figure 2 consolidates the tensile strength data presented in Table 2 into a single visual representation, enabling direct comparison of the three fiber systems across the concentration range studied. The inclusion of error bars (±1 SD, *n* = 5) additionally reveals substantial differences in within-group variability among formulations.

Three observations are noted from Figure 2. First, the rPP–Barley formulation at 10 wt% displays the largest within-group dispersion in the dataset (SD = 1.95 MPa). Second, the rPP–Banana and rPP–Corn curves intersect between 10 and 20 wt%: banana is the highest-performing system at 10 wt% (17.32 MPa) but the lowest at 30 wt% (7.86 MPa), whereas corn maintains a near-plateau response across the concentration range (14.56 → 12.96 MPa). The rank order of the three fiber types is therefore concentration-dependent. Third, none of the biocomposite formulations approaches the tensile strength of the neat rPP control (24.91 MPa). Maximum elongation at break followed a broadly parallel declining trend across all three systems, converging toward comparable low values at 30 wt% (Table 2).

**Figure 3:**
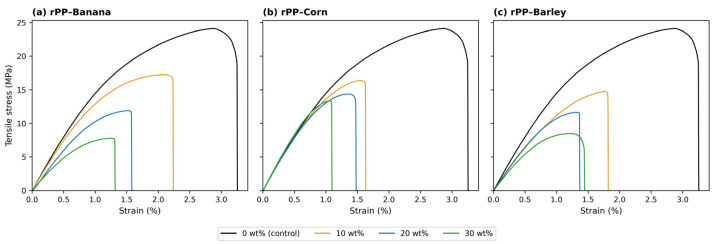
Representative experimental tensile stress–strain curves for (**a**) rPP–Banana, (**b**) rPP–Corn, and (**c**) rPP–Barley biocomposites at 0 (control), 10, 20, and 30 wt% fiber content. For each formulation, the specimen nearest to the group mean in both peak stress and strain at peak stress was selected.

Figure 3 presents representative experimental stress–strain curves for all formulations under tensile loading. For each formulation, the specimen whose peak stress and strain at peak stress were nearest to the group means was selected to illustrate the typical tensile deformation behavior. These curves complement the strength-based comparison shown in Figure 2 by illustrating how fiber type and fiber content modify the deformation response of the recycled polypropylene matrix.

Three deformation features can be identified. First, the strain at peak stress declines progressively with increasing fiber content across all three systems, indicating reduced ductility and a more brittle tensile response. Second, the three rPP–Corn curves remain in close proximity below their peak stresses, reflecting the concentration stability also evident in Figure 2. Third, the elastic modulus of the rPP–Corn system increases monotonically with fiber concentration (Table 2: 1259, 1572, and 1718 MPa at 10, 20, and 30 wt%, respectively) despite the modest decline in peak stress. This stiffness increase produces a visible steepening of the initial slope with fiber content. This stiffness–strength decoupling is consistent with fiber-content-induced stiffening, whereby the increasing lignocellulosic filler fraction rigidifies the composite without enhancing peak load capacity. This pattern is expected when interfacial stress transfer between fiber and matrix is limited by the absence of chemical compatibilization, and it may be practically useful for applications requiring dimensional stability rather than maximum tensile strength.

A two-way ANOVA was performed with fiber type and fiber concentration as independent factors, and their interaction (type × concentration) as a combined term. Table 3 summarizes the results.

Both fiber type (F = 4.775, *p* = 0.013) and fiber concentration (F = 84.559, *p* < 0.001) exerted statistically significant main effects on tensile strength. Concentration dominated the explained sum of squares (SS = 22.677) relative to fiber type (SS = 0.854). The type × concentration interaction did not reach significance at α = 0.05 (F = 2.143, *p* = 0.065).

Tukey’s HSD post hoc test (Table S1, Supplementary Material) indicated that banana fiber composites differed significantly from both corn (*p* = 0.034) and barley (*p* = 0.022), while corn and barley were statistically indistinguishable (*p* = 0.981). Concentration-level comparisons (Table S2, Supplementary Material) showed that all fiber loadings differed significantly from the unfilled control (all *p* < 0.001), and 10 wt% differed significantly from both 20 wt% (*p* = 0.018) and 30 wt% (*p* < 0.001). Critically, 20 wt% and 30 wt% did not differ significantly from each other (*p* = 0.250).

### 3.2. Flexural Properties

Table 4 presents mean flexural strength and maximum flexural strain for neat rPP and all nine biocomposite formulations at 10, 20, and 30 wt% fiber content, with each value representing the mean of five specimens.

The neat rPP control exhibited a flexural strength of 46.25 ± 1.49 MPa with a maximum strain of 5.88%. No biocomposite formulation exceeded this value. The magnitude and pattern of flexural strength reduction varied substantially across fiber types, with inter-system differences more pronounced under flexural than tensile loading.

Banana fiber composites showed the steepest flexural strength decline: 30.87 ± 3.59 MPa at 10 wt% → 22.69 ± 2.16 MPa at 20 wt% → 17.14 ± 1.06 MPa at 30 wt%, representing a monotonic total reduction of 44%. Corn fiber composites exhibited the best absolute flexural retention, declining from 36.60 ± 1.12 MPa at 10 wt% (−21% relative to control) to 24.26 ± 0.95 MPa at 30 wt% (−48%). This represents a steeper decline under flexural than tensile loading, where corn showed only −11% across the full range. Barley fiber composites showed a distinct pattern: flexural strength fell sharply from 32.01 ± 1.30 MPa at 10 wt% to 20.29 ± 1.25 MPa at 20 wt% (−37%), but then partially recovered to 21.94 ± 1.49 MPa at 30 wt% (+8% relative to 20 wt%). This non-monotonic concentration–response is unique among the three fiber systems and was not observed under tensile loading.

Maximum flexural strain declined with fiber incorporation across all systems. One notable exception is barley fiber at 10 wt%, which showed a slightly higher maximum strain than the neat rPP control (6.01% vs. 5.88%).

**Figure 4:**
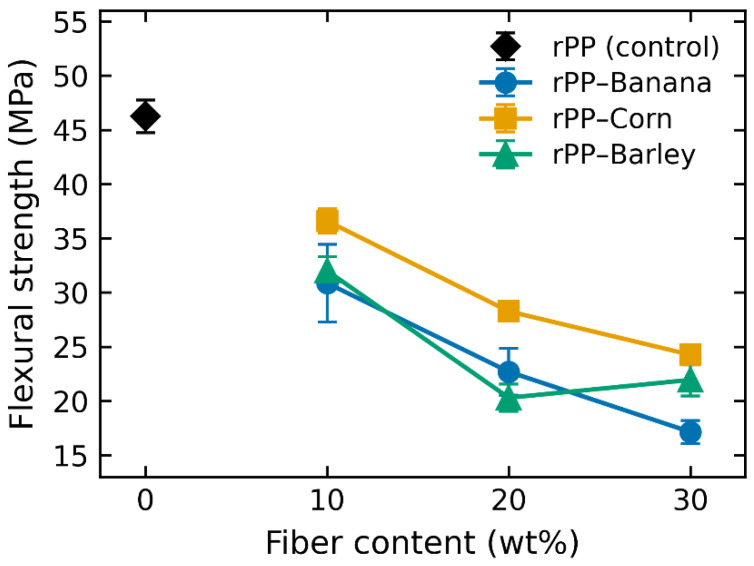
Flexural strength of recycled polypropylene (rPP) and rPP–natural fiber biocomposites as a function of fiber content. Data points represent the mean of five specimens; error bars indicate one standard deviation. The rPP control (filled diamond, 0 wt%) is shown for reference.

Figure 4 consolidates the flexural strength data from Table 4 into a visual comparison of the three fiber systems across the concentration range studied. As in the tensile analysis, error bars (±1 SD, *n* = 5) make within-group variability directly visible.

Four observations are noted from Figure 4. First, the rPP–Corn formulation outperforms both banana and barley systems at all three concentrations, with no curve crossings—a behavior distinct from the tensile case, where banana was the highest-performing system at 10 wt%. Second, the rPP–Banana formulation at 10 wt% displays the largest within-group dispersion in this dataset (SD = 3.59 MPa), in contrast to its low scatter observed under tensile loading. Third, the rPP–Barley system displays a non-monotonic pattern between 20 and 30 wt%, with mean flexural strength remaining essentially flat (20.29 → 21.94 MPa). Fourth, the relative reduction from the neat rPP control to the best-performing biocomposite (corn at 10 wt%) is 21%, smaller than the 30% reduction observed under tensile loading at the same concentration.

**Figure 5:**
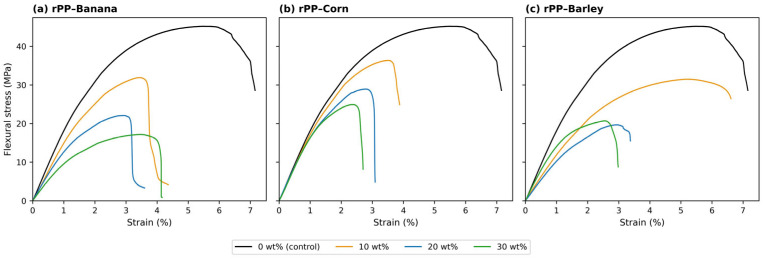
Representative experimental flexural stress–strain curves for (**a**) rPP–Banana, (**b**) rPP–Corn, and (**c**) rPP–Barley biocomposites at 0 (control), 10, 20, and 30 wt% fiber content. For each formulation, the specimen nearest to the group mean in both peak stress and strain at peak stress was selected.

Figure 5 presents representative experimental stress–strain curves for all formulations under three-point flexural loading. For each formulation, the specimen whose peak stress and strain at peak stress were nearest to the group means was selected to illustrate the typical flexural deformation behavior. These curves complement the strength-based comparison shown in Figure 4 by illustrating how fiber type and fiber content modify the flexural response of the recycled polypropylene matrix.

Three deformation features can be identified. First, the rPP–Corn system maintains higher peak stress at all fiber contents relative to the other two systems, confirming the consistent superiority of corn fiber under flexural loading. Second, the rPP–Banana system shows the steepest concentration-dependent reduction in both peak stress and elastic modulus (Table 4: from 1456 to 847 MPa between 10 and 30 wt%), producing progressively flatter and shorter curves consistent with reduced stiffness and limited load transfer at higher fiber contents. Third, the rPP–Barley curves display a non-monotonic concentration response: the 30 wt% curve reaches a slightly higher peak stress than the 20 wt% curve, consistent with the behavior identified in Section 3.2. Additionally, strain at peak stress did not vary substantially across fiber loadings for the banana system (Table 4: 3.45, 3.26, and 3.17% at 10, 20, and 30 wt%, respectively), suggesting that fiber content primarily affects load-bearing capacity rather than deformation extent under flexural loading.

A two-way ANOVA was performed with fiber type and concentration as independent factors, using flexural strength as the dependent variable. Results are presented in Table 5.

All three sources of variation were statistically significant: fiber type (F = 8.872, *p* = 0.001), concentration (F = 98.635, *p* < 0.001), and their interaction (F = 8.331, *p* < 0.001). Concentration dominated the explained sum of squares (SS = 102.838). The type × concentration interaction term (SS = 17.372) is substantially larger relative to the fiber type main effect (SS = 6.166) compared to the tensile ANOVA, where the interaction did not reach significance (*p* = 0.065).

Tukey’s HSD post hoc test by fiber type (Table S3, Supplementary Material) revealed that corn and barley composites differ significantly from each other (*p* = 0.000), while banana composites are not significantly different from either corn (*p* = 0.138) or barley (*p* = 0.071). This pattern is the reverse of that observed under tensile loading, where banana was the statistically distinct system while corn and barley were equivalent.

Concentration-level comparisons (Table S4, Supplementary Material) showed that all pairings involving the unfilled control and the 10 wt% formulations are significant (all *p* < 0.001). The 10 wt% formulation is significantly superior to both 20 wt% (*p* < 0.001) and 30 wt% (*p* < 0.001). Critically, 20 wt% and 30 wt% are not significantly different from each other (*p* = 0.635), the same pattern observed under tensile loading (*p* = 0.250).

### 3.3. Compressive Properties

Table 6 presents mean compressive strength for neat rPP and all nine biocomposite formulations at 10, 20, and 30 wt% fiber content.

The neat rPP control exhibited a compressive strength of 39.67 ± 2.79 MPa. No biocomposite formulation exceeded this value. The magnitude, pattern, and fiber-type specificity of compressive strength reduction differ substantially from the patterns observed under tensile and flexural loading.

Corn fiber composites exhibited the greatest compressive strength retention, declining from 36.56 ± 0.33 MPa at 10 wt% (−8% relative to control) to 35.34 ± 2.26 MPa at 20 wt% (−11%) and 25.84 ± 1.14 MPa at 30 wt% (−35%). The drop between 10 and 20 wt% was only 1.21 MPa (3.3%). Barley fiber composites showed the steepest compressive strength decline of all systems: 29.50 ± 3.85 MPa at 10 wt% (−26%) → 16.89 ± 1.76 MPa at 20 wt% (−57%) → 11.30 ± 0.87 MPa at 30 wt% (−71%). At 30 wt%, barley composites retain only 28% of the control compressive strength, compared to 65% for corn at the same loading—a 2.3-fold gap between the best and worst systems at identical fiber loading. Banana fiber composites showed intermediate behavior, declining monotonically from 32.52 ± 0.65 MPa at 10 wt% (−18%) to 22.75 ± 3.89 MPa at 20 wt% (−43%) and 17.42 ± 5.44 MPa at 30 wt% (−56%).

**Figure 6:**
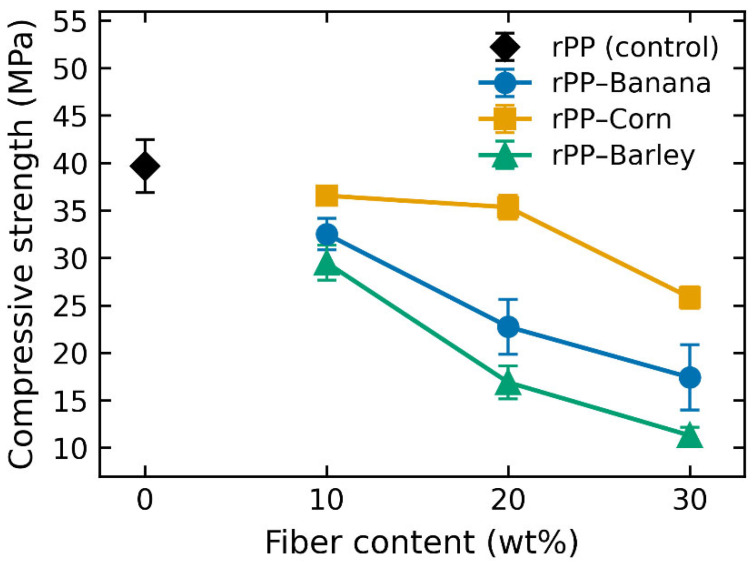
Compressive strength of recycled polypropylene (rPP) and rPP–natural fiber biocomposites as a function of fiber content. Data points represent the mean of five specimens; error bars indicate one standard deviation. The rPP control (filled diamond, 0 wt%) is shown for reference.

Figure 6 consolidates the compressive strength data from Table 6 into a visual comparison of the three fiber systems. As in the previous sections, error bars (±1 SD, *n* = 5) reveal differences in within-group variability that differ markedly from the patterns observed under tensile and flexural loading.

Four observations emerge from Figure 6. First, the gap between the neat rPP control (39.67 MPa) and the best-performing biocomposite (rPP–Corn at 10 wt%, 36.56 MPa) is only 8%, substantially smaller than the corresponding gaps under tensile (30%) and flexural (21%) loading. Second, the rPP–Corn system displays a near-plateau between 10 and 20 wt% (36.56 → 35.34 MPa, a 3.3% drop), combined with the smallest within-group dispersion of any formulation in the entire study (SD = 0.33 MPa at 10 wt%, CV < 1%). This concentration-stable interval (10–20 wt%) differs from the 20–30 wt% plateau identified under tensile and flexural loading. Third, the rank order of the three fiber systems under compression is distinct from both tensile and flexural loading: barley, which occupied an intermediate position under tension and flexion, becomes the weakest system at all concentrations, falling to 11.30 MPa at 30 wt%—the lowest absolute strength recorded in this study. Fourth, the within-group variability of banana composites increases progressively with fiber content (SD = 1.65, 2.89, 3.44 MPa at 10, 20, 30 wt%), a pattern not observed in the other two systems and opposite to the narrowing trend observed for barley under tensile loading (Figure 2).

**Figure 7:**
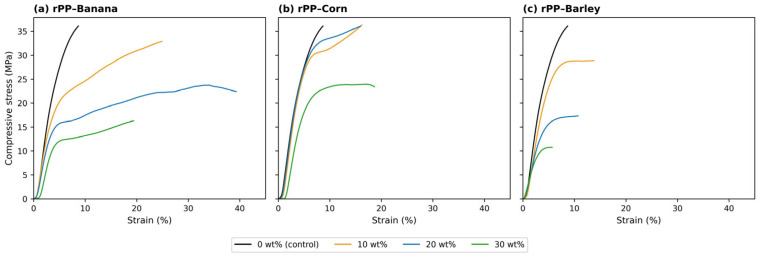
Representative experimental compressive stress–strain curves for (**a**) rPP–Banana, (**b**) rPP–Corn, and (**c**) rPP–Barley biocomposites at 0 (control), 10, 20, and 30 wt% fiber content. For each formulation, the specimen nearest to the group mean in both peak stress and strain at peak stress was selected.

Figure 7 presents representative experimental stress–strain curves for all formulations under compressive loading. For each formulation, the specimen whose peak stress and strain at peak stress were nearest to the group means was selected to illustrate the typical compressive deformation behavior. These curves complement the strength-based comparison shown in Figure 6 by illustrating how fiber type and fiber content modify the compressive response of the recycled polypropylene matrix.

Three deformation features can be identified. First, the near-coincidence of the rPP–Corn 10 and 20 wt% curves throughout most of the loading range, both reaching peak stresses near 35–37 MPa with comparable elastic moduli (Table 6: 789 and 805 MPa, respectively), provides direct visual evidence of the concentration stability identified statistically in Section 3.3 and Section 4.2. Second, the rPP–Banana system displays a progressive reduction in both peak stress and strain at peak stress with increasing fiber content (Table 6: from 32.52 MPa to 17.42 MPa between 10 and 30 wt%), indicating a simultaneous reduction in compressive strength and deformation capacity as fiber loading increases. Third, the rPP–Barley system shows the most severe reduction among the three systems, with the 30 wt% formulation reaching approximately 11 MPa. The contrast between the corn and barley systems highlights the 2.3-fold inter-fiber performance gap at 30 wt%, the largest divergence observed across the mechanical tests in this study.

A two-way ANOVA was performed with fiber type and concentration as independent factors, using compressive strength as the dependent variable. Results are presented in Table 7.

All three sources of variation are highly significant: fiber type (F = 81.231, *p* < 0.001), concentration (F = 182.223, *p* < 0.001), and their interaction (F = 10.111, *p* < 0.001). Concentration dominated the explained sum of squares (SS = 3823.249). The fiber type F-statistic under compression (F = 81.231) is substantially larger than under tension (F = 4.775) and flexion (F = 8.872). The MSE under compression (6.994) is substantially larger than under flexion (0.348), indicating greater within-group variability in the compressive measurements.

Tukey’s HSD post hoc test by fiber type (Table S5, Supplementary Material) showed that all three pairwise comparisons are significant (all *p* < 0.001), establishing a complete ranking: corn > banana > barley. This is the only loading mode in which all three fiber systems are mutually distinguishable by Tukey HSD—under tension, corn and barley were equivalent; under flexion, banana and corn were equivalent.

Concentration-level comparisons (Table S6, Supplementary Material) showed that every pairwise comparison is statistically significant (all *p* < 0.001), including the 20 wt–30 wt% comparison (mean difference = 6.809 MPa, *p* < 0.001). This pattern distinguishes the compressive response from both tensile and flexural behavior, where 20 wt% and 30 wt% were not significantly different (*p* = 0.250 and *p* = 0.635, respectively).

## 4. Discussion

### 4.1. Effect of Fiber Type on Mechanical Performance

The mechanical data presented in Section 3 reveal that fiber type exerts a statistically significant effect on strength under all three loading modes, but that the magnitude and nature of this effect are strongly fiber-specific. The following discussion examines each fiber system in turn, integrating evidence across tensile, flexural, and compressive loading.

Banana pseudostem: Banana fiber composites exhibited the steepest monotonic strength decline with increasing fiber content under all three loading modes: −68% in tension, −44% in flexion, and −56% in compression across the 10–30 wt% range (Table 2, Table 4 and Table 6). This behavior is consistent with reports for uncompatibilized natural fiber–PP systems, where increasing fiber content typically reduces strength due to insufficient fiber wetting and the formation of interfacial defects that act as stress concentrators [7,10]. Banana pseudostem fibers have a relatively high hemicellulose content compared to other lignocellulosic fibers [14,15,16], which has been associated with increased moisture retention and weaker interfacial adhesion in hydrophobic matrices [18,24]. As fiber volume fraction increases, the proportion of the composite volume occupied by the fiber–matrix interface grows, which in uncompatibilized systems is expected to amplify the effect of any such interfacial weakness [7]. Although the present study did not include microstructural characterization to confirm these mechanisms directly, the consistently steeper decline of banana composites relative to corn and barley across all loading modes is consistent with this interpretation. An additional observation is that the within-group variability of banana composites shifts between loading modes: low scatter under tension (SD = 0.21 MPa at 10 wt%) but high scatter under flexion (SD = 3.59 MPa at 10 wt%) and progressively increasing scatter under compression (SD = 1.65 → 2.89 → 3.44 MPa from 10 to 30 wt%). This loading-mode-dependent shift in variability suggests that interfacial defects in banana–rPP composites are activated differently under different stress states, an interpretation that would benefit from microstructural verification in future work. Direct numerical benchmarking of the present banana pseudostem–rPP formulations against published uncompatibilized banana–PP composites is limited by the scarcity of comparable studies: most available literature on banana fiber–PP systems employs MAPP compatibilization, alkaline or silane fiber pretreatment, or both. The banana pseudostem results presented here therefore contribute a reference point for a sparsely documented formulation space.

Corn stover. Corn fiber composites displayed the most favorable mechanical behavior of the three systems across all loading modes. Under tension, the near-flat concentration response (14.56 → 12.96 MPa, only −11% across the full range) contrasts with the behavior reported by Kumar et al. [11], who found that corn fiber–PP composites without MAPP reached peak tensile strength at a fiber fraction as low as 5 wt%, followed by progressive decline. The superior concentration stability observed here suggests that the compression molding processing route—which imposes lower shear stresses than the injection molding used by Kumar et al.—could potentially reduce fiber breakage and preserve fiber geometry, maintaining more uniform load distribution across the matrix volume even at 30 wt% loading, although direct comparison of post-processed fiber morphology between the two methods was not performed. Under flexion, corn retained the best absolute strength at all concentrations (Figure 4), with no rank-order crossings—unlike the tensile case where banana outperformed corn at 10 wt%. Kumar et al. [11] reported that corn fiber–PP composites without MAPP reached peak flexural strength at only 5 wt%; the 10 wt% formulation in the present study achieves a higher absolute flexural strength retention, a difference that may reflect the lower processing shear inherent to hot-press molding. Under compression, corn exhibited a near-plateau between 10 and 20 wt% (36.56 → 35.34 MPa, 3.3% drop) with the smallest within-group dispersion of the entire study (SD = 0.33 MPa, CV < 1%). The compact morphology of corn fibers, consistent with the observations of Bledzki et al. [13] regarding particle dispersion and morphology of analogous cereal residue fillers, may resist agglomeration under processing conditions, distributing stress more uniformly through the matrix volume. As a numerical reference, Flandez et al. [32] reported a tensile strength of 21.05 MPa for uncompatibilized corn stalk flour–PP composites at 40 wt% loading under injection molding. The lower values in the present system (12.96 MPa at 30 wt%) are consistent with the use of recycled rather than virgin PP as matrix, and with the lower consolidation pressure achievable under contact hot-press molding relative to injection molding. The qualitative trend—monotonic decline with fiber addition in uncompatibilized systems—is consistent between the two studies.

Barley fiber composites exhibited the most loading-mode-sensitive behavior of the three systems. Under tension, the response was intermediate (−36% across the full range), with comparatively narrow standard deviations at certain concentrations suggesting more uniform fiber dispersion. Bledzki et al. [13] characterized barley husk particles as predominantly round and angular in shape, with 75–80% of particles distributed in the 50–300 μm size range, and noted that the fiber surface is coated with fat and protein molecules. This surface chemistry—though reducing chemical compatibility with PP—may limit inter-fiber agglomeration by acting as a physical barrier between adjacent fibers, partially explaining the more homogeneous mechanical response observed at intermediate tensile loadings. Under flexion, barley showed a unique non-monotonic concentration–response: strength fell sharply from 32.01 to 20.29 MPa between 10 and 20 wt% but partially recovered to 21.94 MPa at 30 wt%. One possible explanation is that increased fiber–fiber contact at higher packing fractions generates a degree of physical constraint within the matrix that partially offsets the weak interfacial adhesion. However, alternative explanations—including changes in void distribution or fiber orientation with packing fraction—cannot be ruled out without microstructural examination, which is identified as a priority for future work. Under compression, barley showed the most severe decline of any system (−71% at 30 wt%), with the lowest absolute strength recorded in the study (11.30 MPa). The fat and protein surface coating documented by Bledzki et al. [13] impairs interfacial wetting by the non-polar PP matrix due to the low surface energy of these lipids; while this coating may partially limit inter-fiber agglomeration under flexion (as suggested by the non-monotonic response), under compressive loading the resulting poor matrix–fiber contact appears to create a high density of stress-concentrating voids that propagate rapidly as fiber content increases. Morales et al. [18] similarly reported that poor interfacial bonding between untreated rice husk and rPP generates interfacial debonding and void formation acting as stress concentrators—a mechanism directly applicable to the barley husk system. The 2.3-fold performance gap between corn (25.84 MPa) and barley (11.30 MPa) at 30 wt% is the largest inter-fiber divergence observed across all three mechanical tests and underscores that fiber type selection is critically important under compressive loading.

### 4.2. Effect of Fiber Concentration and the 20 wt% Performance Plateau

Across all three loading modes, fiber concentration was the dominant statistical driver of mechanical performance, consistently accounting for the largest share of the explained sum of squares: SS = 22.677 under tension, SS = 102.838 under flexion, and SS = 3823.249 under compression (Table 3, Table 5 and Table 7). This dominance of concentration over fiber type is consistent with the findings of Kumar et al. [11] and Bledzki et al. [13], both of whom reported fiber weight fraction as the primary control variable for strength in PP composites with lignocellulosic fillers. The practical implication is that, for uncompatibilized rPP–lignocellulosic systems, the decision of how much fiber to incorporate has a greater impact on mechanical performance than the decision of which fiber to use—although the latter becomes increasingly important under compressive loading, where the fiber type F-statistic reaches F = 81.231 (Section 4.1).

A central finding of this study is the identification of a performance plateau at approximately 20 wt% fiber content under both tensile and flexural loading. Tukey HSD post hoc comparisons showed that 20 wt% and 30 wt% formulations did not differ significantly from each other under tension (*p* = 0.250) or flexion (*p* = 0.635), while all other adjacent concentration pairs were significantly different (Tables S2 and S4). This convergence is reinforced by the dominance of concentration as the largest contributor to explained variance in both ANOVA models (Table 3 and Table 5), supporting the interpretation of the plateau as a robust composition-level finding rather than an artifact of a particular loading geometry. The convergence of this threshold across two independent loading modes is, to the authors’ knowledge, a finding that has not been previously reported for uncompatibilized natural fiber–rPP systems. This may reflect the fact that prior studies have generally reported mean strength values without testing pairwise statistical differences between adjacent concentration levels, which would obscure the existence of such a plateau even where one is present in the underlying data.

The practical significance of this plateau is that fiber content can be increased from 20 to 30 wt%—a 50% increase in filler mass fraction—without statistically detectable loss in tensile or flexural strength. For cost-driven or waste-valorization-driven applications, where maximizing fiber incorporation is desirable to reduce polymer consumption or to absorb larger volumes of agro-industrial residue, this plateau defines a formulation window in which additional fiber loading imposes no measurable mechanical penalty under tensile and flexural service conditions.

Under compressive loading, however, no such shared plateau exists. Every pairwise concentration comparison was statistically significant (all *p* < 0.001, Table S6), including the 20 wt–30 wt% pair (mean difference = 6.809 MPa). Compressive strength continues to decline significantly at every concentration increment across the full range tested. This absence of a concentration threshold under compression means that, for applications where compressive load bearing is primary, each increment in fiber loading carries a measurable performance penalty with no saturation effect—in contrast to the formulation flexibility available under tensile and flexural conditions.

An additional observation is that the concentration-stable interval identified for the corn fiber system under compression (10–20 wt%, a 3.3% drop; Section 3.3, Figure 6) occurs at a different range than the shared 20–30 wt% plateau observed under tension and flexion. To the authors’ knowledge, this loading-mode-dependent shift in the plateau interval has not been previously documented. The observation suggests that the fiber concentration at which further addition ceases to affect strength is not a fixed material property but rather depends on the dominant failure mechanism active under each stress state. Under tension and flexion, where interfacial debonding governs failure, the plateau may correspond to the saturation of interfacial defect density. Under compression, where matrix shear yielding, particle–matrix debonding, and void nucleation around poorly bonded inclusions govern failure [7,18], the concentration sensitivity may persist to higher loadings because the volumetric distribution of voids and agglomerates continues to evolve with each increment in filler fraction. However, the mechanistic origin of these convergent and divergent thresholds cannot be established from the present mechanical data alone and warrants targeted investigation, including microstructural characterization and finer concentration sampling between 10 and 30 wt%, in future work.

### 4.3. Loading-Mode-Dependent Behavior

The observed mechanical degradation is consistent with limited interfacial adhesion between hydrophilic lignocellulosic fibers and hydrophobic rPP, as widely reported in uncompatibilized systems [7,12]. However, the distinct loading-mode-dependent behavior documented in Section 3 suggests that interfacial effects alone do not fully explain the mechanical response. If adhesion governed performance uniformly, fiber type effects should scale similarly across stress states—yet the data show otherwise.

A central finding of this study is that the relative performance of the three fiber systems is not constant across loading modes—the rank order, the degree of statistical differentiation, and the variability patterns all change depending on whether the composite is tested under tension, flexion, or compression. This loading-mode dependence has practical consequences for material specification and argues against the common practice of characterizing biocomposite formulations under a single loading mode.

The most direct evidence for loading-mode-dependent behavior is the progressive increase in the fiber type F-statistic across the three tests: F = 4.775 under tension, F = 8.872 under flexion, and F = 81.231 under compression (Table 3, Table 5 and Table 7). This nearly 17-fold increase from tension to compression indicates that the three fiber systems become increasingly differentiated as the dominant stress state changes. This finding is contrary to what might be expected from the conventional assumption that interfacial adhesion—and therefore fiber type effects—are less critical under compression than under tension [7]. Instead, the data suggest that the microstructural factors governing compressive failure—which may include void density, agglomerate structure, and fiber packing characteristics—are more strongly fiber-type dependent than the interfacial adhesion parameters that dominate tensile failure. The contrasting surface compositions and structural architectures reported for different agricultural by-products [13,18] are consistent with this interpretation.

The type × concentration interaction provides a second line of evidence. Under tension, this interaction was borderline (F = 2.143, *p* = 0.065), suggesting that concentration effects were broadly consistent across fiber types within the statistical power of the design. Under flexion, the interaction reached unambiguous significance (F = 8.331, *p* < 0.001), confirming that the concentration–response is genuinely fiber-type specific under bending. Under compression, the interaction was also significant (F = 10.111, *p* < 0.001). The progression from borderline to significant interaction across loading modes confirms that bending and compression are more discriminating tests for differentiating biocomposite formulations than tension—a finding with methodological implications for future studies of uncompatibilized systems, which should include at least two loading modes to capture the full spectrum of fiber type effects.

The Tukey HSD groupings further illustrate the loading-mode dependence. Under tension, banana was the statistically distinct system while corn and barley were equivalent (Table S1). Under flexion, the grouping reversed: corn and barley were mutually distinguishable while banana overlapped with both (Table S3). Under compression, all three systems were completely separated (Table S5)—the only loading mode achieving full statistical discrimination. This progressive refinement of the Tukey groupings—from two groups under tension, to a partially overlapping pattern under flexion, to three completely distinct groups under compression—reinforces the conclusion that compressive loading is the most discriminating test for characterizing fiber-type effects in these uncompatibilized rPP biocomposites.

The rank order itself also shifts. Under tension, the rank at 10 wt% is banana > barley ≈ corn, but at 30 wt%, it becomes corn > barley > banana (Figure 2). Under flexion, corn maintains the top position at all concentrations (Figure 4). Under compression, corn again leads but barley—which occupied an intermediate position under tension and flexion—drops to the weakest system at all concentrations (Figure 6). The barley system therefore exhibits the most loading-mode-sensitive behavior of the three: intermediate under tension, non-monotonic under flexion, and dramatically inferior under compression. As discussed in Section 4.1, this sensitivity likely reflects the specific interplay between the fat and protein surface coating of barley husk fibers [13] and the dominant failure mechanism active under each stress state.

Variability patterns add a further dimension to the loading-mode dependence. The fiber system displaying the largest within-group dispersion shifts between loading modes: barley at 10 wt% under tension (SD = 1.95 MPa), banana at 10 wt% under flexion (SD = 3.59 MPa), and banana at 30 wt% under compression (SD = 3.44 MPa). These shifts indicate that the specimen-to-specimen heterogeneity in mechanical response is not an intrinsic property of a given fiber type but depends on how microstructural defects are activated under different stress states. One possible interpretation is that compressive failure is more sensitive to localized microstructural heterogeneities such as void clusters and agglomerates than tensile or flexural failure, as reflected in the substantially larger MSE under compression (6.994) compared to flexion (0.348) and tension (0.089). If void clusters or filler agglomerates are present—as reported for analogous uncompatibilized rPP–lignocellulosic systems [18]—their spatially variable distribution from specimen to specimen could account for the greater scatter observed under compression. This interpretation would benefit from microstructural verification in future work.

Taken together, the loading-mode-dependent patterns documented here—in fiber type F-statistics, interaction significance, Tukey groupings, rank orders, and variability—establish that mechanical characterization under a single loading mode provides an incomplete and potentially misleading picture of biocomposite performance. The data support the adoption of multi-modal testing protocols as standard practice for uncompatibilized natural fiber–rPP systems, particularly when the target application involves combined or variable stress states.

### 4.4. Benchmarking with the Literature

Contextualizing the mechanical performance of the present formulations within the broader literature requires consideration of three systematic differences between this study and most published work on natural fiber–PP composites: (i) the use of recycled rather than virgin polypropylene as matrix, (ii) the use of contact hot-press molding at ~45 kPa rather than conventional injection molding or matched-die compression at 2–20 MPa, and (iii) the complete absence of compatibilizer or fiber pretreatment. Each of these factors is expected to reduce absolute mechanical performance relative to systems employing virgin PP, higher consolidation pressures, or MAPP coupling. The relevant comparison is therefore not whether the present values match or exceed those in the literature, but whether they fall within a range consistent with the combined effect of these three constraints.

The tensile strengths measured across the nine biocomposite formulations (7.86–17.32 MPa) fall within the broader range of values reported by Sobczak et al. [33] in their comprehensive survey of PP–natural fiber composites processed without compatibilizers. This indicates that the present formulations, although below the strength of the neat rPP control, are representative of the baseline performance expected for uncompatibilized PP–lignocellulosic systems. For corn stover specifically, Flandez et al. [32] reported a tensile strength of 21.05 MPa for uncompatibilized corn stalk flour–PP composites at 40 wt% under injection molding, using mechanically milled corn stalk flour—a preparation analogous to the raw corn stover used here. The lower values in the present system (12.96 MPa at 30 wt%) are quantitatively consistent with the combined effect of matrix degradation in recycled PP and the substantially lower consolidation pressure of the hot-press process. The qualitative trend—a monotonic decline in tensile strength with increasing fiber content in uncompatibilized systems—is consistent between the two studies. For banana pseudostem, direct numerical benchmarking is constrained by the scarcity of comparable studies employing uncompatibilized banana fiber in PP matrices: most available literature employs MAPP compatibilization, alkaline or silane fiber pretreatment, or both [14,15,16]. For barley husk, Bledzki et al. [13] reported mechanical properties for barley husk–PP composites with coupling agents, achieving tensile strengths substantially above those observed here—a difference attributable primarily to the absence of MAPP in the present formulations.

Under flexural loading, the same systematic constraints apply. The neat rPP flexural strength (46.25 MPa) and the biocomposite range (17.14–36.60 MPa) are consistent with values reported for uncompatibilized natural fiber–PP systems in the general literature [7,17]. The observation that corn retains the best absolute flexural strength at all concentrations is consistent with the favorable concentration stability reported by Kumar et al. [11] for corn–PP systems at low fiber fractions, although Kumar’s study employed injection molding and reported peak flexural performance at only 5 wt% fiber content. The higher retention observed in the present study at 10 wt% may reflect the lower processing shear inherent to hot-press molding, which could preserve fiber geometry more effectively than injection molding, although direct comparison of post-processed fiber morphology was not performed.

Under compressive loading, benchmarking is more limited because, as noted by Faruk et al. [17], the literature on natural fiber–PP composites has historically focused on tensile, flexural, and impact properties, with compressive behavior receiving less systematic attention. The compressive strengths reported here (11.30–36.56 MPa for biocomposites, 39.67 MPa for neat rPP) therefore contribute to an underdocumented property space. The observation that corn fiber at 10 wt% retains 92% of the neat rPP compressive strength—compared to 70% retention under flexion and 58% under tension—suggests that compressive applications represent the most favorable loading scenario for these uncompatibilized formulations, a finding that has not been widely discussed in the biocomposite literature.

It is worth noting that the processing conditions used in this study—single-screw extrusion at 172 °C followed by contact hot-press molding at ~45 kPa—were deliberately selected to minimize thermal degradation of the lignocellulosic phase. The barrel temperature of 172 °C is well below the conventional processing window for virgin polypropylene (220–280 °C) [23] and below the threshold at which lignocellulosic fibers begin to undergo measurable thermal degradation (~200 °C, with hemicellulose being the most thermally labile component) [24]. In PP–cellulose fiber composites, Feldmann [25] demonstrated that melt temperatures above approximately 256 °C cause measurable fiber length reduction and loss of mechanical properties. While these conservative processing conditions protect fiber integrity, they also limit melt viscosity reduction and consolidation pressure, contributing to the lower absolute strength values relative to injection-molded systems reported in the literature. The trade-off between fiber preservation and consolidation quality is an inherent constraint of low-pressure, low-temperature processing routes for lignocellulosic biocomposites and should be considered when interpreting cross-study comparisons.

### 4.5. Practical Implications

The mechanical envelope established in this study suggests three categories of practical application for these uncompatibilized rPP biocomposites.

For components requiring moderate flexural strength and dimensional stability under bending—such as packaging inserts, decorative panels, or non-structural automotive trims—rPP–Corn formulations at 10–20 wt% offer 61–79% of the neat rPP flexural strength with a corresponding 10–20 wt% mass-fraction replacement of the petroleum-derived rPP matrix by agro-industrial residue. The corn fiber system is particularly favorable for these applications because its elastic modulus is the highest among the three fiber systems tested at all evaluated concentrations under flexural loading (Table 4), providing the relative stiffness retention required for dimensional stability under load.

For compressive-dominated applications such as low-load pallets, dunnage, or impact-absorbing inserts, rPP–Corn at 10 wt% retains 92% of the neat rPP compressive strength (36.56 vs. 39.67 MPa) and exhibits the smallest within-group variability of the entire study (SD = 0.33 MPa). This represents the most favorable loading scenario for raw fiber incorporation and the strongest case for direct industrial deployment without further interfacial modification.

For cost-driven applications where mechanical performance is secondary to material substitution rate, the 20 wt% formulation under tensile and flexural service conditions provides a formulation window where additional fiber loading up to 30 wt% does not impose statistically detectable strength penalties (Section 4.2). Under these conditions, the substitution rate of virgin polymer can be increased from 20 to 30 wt% without measurable loss of mechanical performance—a property profile particularly relevant for cost-sensitive applications in agro-industrial regions where the residue feedstocks are locally available at near-zero cost.

### 4.6. Limitations and Future Work

Several limitations of the present study should be acknowledged, as they define the boundaries within which the reported findings can be interpreted and identify priorities for future investigation.

First, no microstructural characterization (SEM, FTIR, TGA, XRD) was performed on either the raw fibers or the fracture surfaces of the tested specimens. The mechanistic interpretations offered in Section 4.1, Section 4.2 and Section 4.3—including the roles attributed to hemicellulose content, fat and protein surface coatings, void nucleation, and fiber agglomeration—are therefore based on analogies with published systems rather than on direct observation of the present materials. Future work should include scanning electron microscopy of fracture surfaces under all three loading modes to verify whether the failure mechanisms inferred from the mechanical data (interfacial debonding under tension, matrix shear yielding and void coalescence under compression) are consistent with the morphological evidence.

Second, the consolidation pressure achievable with the open-cavity hot-press mold used in this study (~45 kPa) is substantially below the range typical of matched-die compression molding (2–20 MPa) and injection molding (>50 MPa). This low pressure may result in incomplete fiber wetting, higher void content, and reduced matrix consolidation relative to conventional processing routes, contributing to the lower absolute strength values discussed in Section 4.4. While the contact hot-press approach was selected for its simplicity and accessibility in the context of agro-industrial residue valorization, quantifying the void fraction and comparing it with injection-molded equivalents would strengthen the interpretation of the mechanical data.

Third, mechanical tests were performed on five specimens per condition (*n* = 5). While this sample size is sufficient for the detection of the large main effects observed (concentration effects consistently reached *p* < 0.001), the type × concentration interaction under tensile loading approached but did not reach significance (*p* = 0.065), and a study with larger replication might resolve this borderline result. Similarly, the within-group variability patterns discussed in Section 4.3—particularly the loading-mode-dependent shifts in scatter—would benefit from larger sample sizes to confirm their robustness.

Fourth, the study evaluated only tensile, flexural, and compressive static properties. Impact resistance (Charpy or Izod), dynamic mechanical analysis (DMA), and long-term properties such as creep and moisture absorption were not assessed. For non-structural applications where these biocomposites are most likely to be deployed, impact and moisture performance may be equally or more important than static strength, and their evaluation is recommended for future work.

Fifth, a single particle size fraction (1–2 mm, retained between M10 and M18 sieves) was used for all three fiber types. The influence of particle size on the observed fiber-type differences, concentration thresholds, and loading-mode-dependent behavior remains unknown. Finer concentration sampling between 10 and 30 wt%—particularly in the 15–25 wt% range—combined with multiple particle size fractions, would enable more precise identification of the plateau thresholds documented in Section 4.2 and clarification of their mechanistic origin.

Sixth, the mechanistic origin of the 20 wt% performance plateau observed under tensile and flexural loading, and its absence under compression, remains an open question. As discussed in Section 4.2, the convergence of this threshold across two loading modes suggests a structural rather than coincidental origin, but targeted investigation—including microstructural characterization of specimens at 15, 20, and 25 wt% and finer statistical resolution through increased replication—is needed to establish whether the plateau reflects saturation of interfacial defect density, a percolation threshold in filler connectivity, or another mechanism.

Finally, compatibility enhancement through MAPP coupling or alkaline fiber pretreatment was deliberately excluded from the present study in order to establish an uncompatibilized baseline. The performance gap between the values reported here and those achievable with compatibilization—as documented for corn stover by Flandez et al. [32] and for barley husk by Bledzki et al. [13]—defines the improvement margin available through interfacial modification and represents a natural next step for this research program.

## 5. Conclusions

This study establishes the mechanical performance envelope of uncompatibilized rPP biocomposites filled with raw banana pseudostem, corn stover, and barley husk fibers at 10, 20, and 30 wt% under tensile, flexural, and compressive loading. The following conclusions are supported by the experimental data:

1. Fiber concentration is the dominant driver of mechanical performance across all three loading modes, consistently accounting for the largest share of explained variance in the ANOVA models. Tensile strength declined monotonically with fiber loading across all systems, with banana composites experiencing the largest reduction (−68% at 30 wt%) and corn composites exhibiting exceptional concentration stability (−11% reduction between 10 and 30 wt% loadings).
2. Under flexural loading, corn fiber composites achieved the best absolute performance retention at all concentrations (36.60 MPa at 10 wt%, −21% relative to neat rPP). The type × concentration interaction was unambiguously significant under flexion (F = 8.331, *p* < 0.001) but only borderline under tension (*p* = 0.065), confirming that bending is the most discriminating loading mode for revealing fiber-type-specific concentration responses. Barley fiber exhibited a unique non-monotonic flexural response, with partial strength recovery between 20 and 30 wt%, not observed under any other loading condition or fiber system.
3. Compressive loading was the mode most strongly differentiated by fiber type: the fiber type F-statistic reached F = 81.231—nearly 17-fold higher than under tension—and all three fiber systems were mutually distinguishable by Tukey HSD, the only loading mode achieving complete statistical separation. The 2.3-fold performance gap between corn (25.84 MPa) and barley (11.30 MPa) at 30 wt% is the largest inter-fiber divergence observed across all tests, indicating that fiber type selection is most mechanically consequential under compressive loading.
4. A performance plateau at approximately 20 wt% was identified under both tensile (*p* = 0.250) and flexural (*p* = 0.635) loading: the 20–30 wt% increment produced no statistically significant strength reduction in either mode. This plateau does not extend to compressive loading, where all concentration increments produced significant reductions (all *p* < 0.001). The convergence of this threshold across two loading modes, and its absence under a third, has not been previously reported for uncompatibilized natural fiber–rPP systems.
5. Collectively, these results demonstrate that mechanical characterization under a single loading mode provides an incomplete picture of biocomposite performance. Corn fiber composites at 10–20 wt% emerge as the most versatile formulation across all loading modes tested, and the loading-mode-resolved mechanical baseline established here supports the technical viability of uncompatibilized rPP biocomposites—formulated entirely from post-consumer plastic and local agro-industrial residues—for non-structural applications.
6. Future research should prioritize (i) SEM-EDS analysis of fracture surfaces to verify the inferred failure mechanisms; (ii) evaluation of impact resistance, creep, and moisture absorption relevant to non-structural applications; (iii) systematic comparison with MAPP-compatibilized formulations to quantify the improvement margin attainable through interfacial modification; and (iv) finer concentration sampling between 10 and 30 wt% to characterize the plateau thresholds. Subsequent stages of this research line are directed toward the applied development of biocomposite-based non-structural construction components, with translation of the resulting outcomes to industrial partners in the regional construction sector.

## Figures and Tables

**Table 1:** Specimen dimensions according to ASTM standards.

Test	Standard	Geometry	Key Dimensions
Tensile	ASTM D638-03	Dog-bone (Type IV)	Thickness: 3.2 mm; Width: 19 mm; Length: 115 mm
Flexural	ASTM D790-03	Rectangular bar	Thickness: 3.2 mm; Width: 12.7 mm; Length: 125 mm
Compressive	ASTM D695-02a	Cylinder	Length: 25.4 mm; Diameter: 12.7 mm

**Table 2:** Tensile properties of rPP and rPP–fiber biocomposites (mean ± SD, *n* = 5).

Composite	Fiber Type	Fiber Content (wt%)	Tensile Strength (MPa)	Elastic Modulus (MPa)	Max. Elongation (%)
rPP	—	0	24.91 ± 0.72	1577 ± 24	2.75
rPP–Banana	Banana	10	17.32 ± 0.21	1523 ± 31	2.04
		20	11.69 ± 0.41	1216 ± 35	1.57
		30	7.86 ± 0.75	940 ± 85	1.17
rPP–Corn	Corn	10	14.56 ± 0.42	1259 ± 67	1.43
		20	13.96 ± 0.71	1572 ± 70	1.39
		30	12.96 ± 1.30	1718 ± 117	0.97
rPP–Barley	Barley	10	14.61 ± 1.95	1380 ± 34	1.63
		20	10.21 ± 1.02	1268 ± 35	1.38
		30	9.33 ± 0.73	1151 ± 86	1.24

**Table 3:** Two-way ANOVA results for tensile strength.

Source	SS	df	MS	F	*p*-Value
Fiber type	0.854	2	0.427	4.775	0.013
Concentration	22.677	3	7.559	84.559	0.000
Type × Concentration	1.149	6	0.192	2.143	0.065
Error	4.291	48	0.089	—	—
Total	204.709	60	—	—	—

**Table 4:** Flexural properties of rPP and rPP–natural fiber biocomposites (mean ± SD, *n* = 5).

Composite	Fiber Type	Fiber Content (wt%)	Flexural Strength (MPa)	Elastic Modulus (MPa)	Max. Flexural Strain (%)
rPP	—	0	46.25 ± 1.49	1710 ± 55	5.88
rPP–Banana	Banana	10	30.87 ± 3.59	1456 ± 99	3.45
		20	22.69 ± 2.16	1259 ± 31	3.26
		30	17.14 ± 1.06	847 ± 42	3.17
rPP–Corn	Corn	10	36.60 ± 1.12	1552 ± 54	3.86
		20	28.27 ± 0.79	1698 ± 49	2.51
		30	24.26 ± 0.95	1654 ± 119	2.05
rPP–Barley	Barley	10	32.01 ± 1.30	1049 ± 71	6.01
		20	20.29 ± 1.25	973 ± 107	2.88
		30	21.94 ± 1.49	1428 ± 37	2.67

**Table 5:** Two-way ANOVA results for flexural strength.

Source	SS	df	MS	F	*p*-Value
Fiber type	6.166	2	3.083	8.872	0.001
Concentration	102.838	3	34.279	98.635	0.000
Type × Concentration	17.372	6	2.895	8.331	0.000
Error	16.682	48	0.348	—	—
Total	1082.979	60	—	—	—

**Table 6:** Compressive properties of rPP and rPP–natural fiber biocomposites (mean ± SD, *n* = 5).

Composite	Fiber Type	Fiber Content (wt%)	Compressive Strength (MPa)	Elastic Modulus (MPa)	Max. Compressive Strain (%)
rPP	—	0	39.67 ± 2.79	1145 ± 67	7.94
rPP–Banana	Banana	10	32.52 ± 0.65	674 ± 15	26.28
		20	22.75 ± 3.89	570 ± 8	29.37
		30	17.42 ± 5.44	475 ± 6	18.13
rPP–Corn	Corn	10	36.56 ± 0.33	789 ± 39	15.57
		20	35.34 ± 2.26	805 ± 37	15.20
		30	25.84 ± 1.14	585 ± 34	18.33
rPP–Barley	Barley	10	29.50 ± 3.85	647 ± 18	13.59
		20	16.89 ± 1.76	489 ± 35	9.93
		30	11.30 ± 0.87	401 ± 42	5.46

**Table 7:** Two-way ANOVA results for compressive strength.

Source	SS	df	MS	F	*p*-Value
Fiber type	1136.212	2	568.106	81.231	0.000
Concentration	3823.249	3	1274.416	182.223	0.000
Type × Concentration	424.268	6	70.711	10.111	0.000
Error	335.698	48	6.994	—	—
Total	55,645.789	60	—	—	—

## Data Availability

The original contributions presented in this study are included in the article/Supplementary Material. Further inquiries can be directed to the corresponding author.

## Supplementary Materials

The following supporting information can be downloaded at: https://www.mdpi.com/article/10.3390/polym18111384/s1, Table S1: Tukey HSD post-hoc pairwise comparisons for tensile strength by fiber type. Mean differences marked with * are significant at α = 0.05; Table S2: Tukey HSD post-hoc pairwise comparisons for tensile strength by fiber concentration. Mean differences marked with * are significant at α = 0.05; Table S3: Tukey HSD post-hoc pairwise comparisons for flexural strength by fiber type. Mean differences marked with * are significant at α = 0.05; Table S4: Tukey HSD post-hoc pairwise comparisons for flexural strength by fiber concentration. Mean differences marked with * are significant at α = 0.05; Table S5: Tukey HSD post-hoc pairwise comparisons for compressive strength by fiber type. Mean differences marked with * are significant at α = 0.05; Table S6: Tukey HSD post-hoc pairwise comparisons for compressive strength by fiber concentration. Mean differences marked with * are significant at α = 0.05.
