# Supplementary material for: Mechanical Performance of Uncompatibilized Recycled Polypropylene Biocomposites Filled with Corn, Banana, and Barley Agro-Industrial Residue Fibers
Source: Polymers (Basel). 2026 Jun 2;18(11):1384. doi: 10.3390/polym18111384 (PMC13259176; doi:10.3390/polym18111384)
Supplement: Supplementary file 1 [file polymers-18-01384-s001.zip › polymers-4328322-supplementary.pdf]

polymers

MDPI

Supplementary Materials

Mechanical Performance of Uncompatibilized Recycled Polypropylene Biocomposites Filled with Corn, Banana, and Barley Agro-industrial Residue Fibers

Juan Fernando García [1,*] and Juan Diego Febres [1]

[1] Departamento de Química y Producción, Universidad Técnica Particular de Loja, Loja 110107, Ecuador
* Correspondence: jfgarcia@utpl.edu.ec

Table S1. Tukey HSD post-hoc pairwise comparisons for tensile strength by fiber type. Mean differences marked with * are significant at α = 0.05.

Comparison (I)	Comparison (J)	Mean Difference (I–J)	p-value
rPP–Banana	rPP–Corn	0.243703*	0.034
	rPP–Barley	0.261418*	0.022
rPP–Corn	rPP–Banana	−0.243703*	0.034
	rPP–Barley	0.017715	0.981
rPP–Barley	rPP–Banana	−0.261418*	0.022
	rPP–Corn	−0.017715	0.981

Table S2. Tukey HSD post-hoc pairwise comparisons for tensile strength by fiber concentration. Mean differences marked with * are significant at α = 0.05.

Comparison	Mean Difference	p-value
0 wt% – 10 wt%	1.057097*	0.000
0 wt% – 20 wt%	1.391267*	0.000
0 wt% – 30 wt%	1.596595*	0.000
10 wt% – 20 wt%	0.334170*	0.018
10 wt% – 30 wt%	0.539497*	0.000
20 wt% – 30 wt%	0.205327	0.250

Table S3. Tukey HSD post-hoc pairwise comparisons for flexural strength by fiber type. Mean differences marked with * are significant at α = 0.05.

Comparison (I)	Comparison (J)	Mean Difference (I–J)	p-value
rPP–Banana	rPP–Corn	0.36241	0.138
	rPP–Barley	-0.4221	0.071
rPP–Corn	rPP–Banana	-0.3624	0.138
	rPP–Barley	-0.784512*	0.000

rPP–Barley	rPP–Banana	0.42211	0.071
	rPP–Corn	0.784512*	0.000

Table S4. Tukey HSD post-hoc pairwise comparisons for flexural strength by fiber concentration. Mean differences marked with * are significant at α = 0.05.

Comparison	Mean Difference	p-value
0 wt% – 10 wt%	1.441769*	0.000
0 wt% – 20 wt%	2.997715*	0.000
0 wt% – 30 wt%	3.253915*	0.000
10 wt% – 20 wt%	1.555946*	0.000
10 wt% – 30 wt%	1.812145*	0.000
20 wt% – 30 wt%	0.256199	0.635

Table S5. Tukey HSD post-hoc pairwise comparisons for compressive strength by fiber type. Mean differences marked with * are significant at α = 0.05.

Comparison (I)	Comparison (J)	Mean Difference (I–J)	p-value
rPP–Banana	rPP–Corn	-6.428425*	0.000
	rPP–Barley	4.149375*	0.000
rPP–Corn	rPP–Banana	6.428425*	0.000
	rPP–Barley	10.577800*	0.000
rPP–Barley	rPP–Banana	-4.149375*	0.000
	rPP–Corn	-10.577800*	0.000

Table S6. Tukey HSD post-hoc pairwise comparisons for compressive strength by fiber concentration. Mean differences marked with * are significant at α = 0.05.

Comparison	Mean Difference	p-value
0 wt% – 10 wt%	6.493253*	0.000
0 wt% – 20 wt%	14.354620*	0.000
0 wt% – 30 wt%	21.164013*	0.000
10 wt% – 20 wt%	7.861367*	0.000
10 wt% – 30 wt%	14.670760*	0.000
20 wt% – 30 wt%	6.809393*	0. 000
